# Tin Foil

**Published:** 1871-07

**Authors:** L. D. Walter

**Affiliations:** Rochester, N. Y.


### ARTICLE X.

### *Tin Foil.*

By L. D. Walter, D.D.S., Rochester, N. Y.

It is very seldom, indeed, that anything is said in the *Dental Journals* concerning the history and properties of this (to the dentists) almost indispensible article. Its history, as a metal, is given in detail in all works of metallurgy, and, as a filling, in the two or three works now extant on operative dentistry. Its properties are mentioned and dilated upon more or less completely in the above works, but the one to which this article would call attention—the property of cohesiveness—is set aside and numbered with those deserving of little, if any, consideration. That tin foil is cohesive to quite a marked degree is not generally known; a fact attributable partly to faulty manipulations, and partly to the scarcity of a pure article properly made. This cohesive property is seldom recognized (for the reason above stated), though it is often ignorantly made use of by old and young operators.

In tin foil, as well as in gold, it is found best developed in the pure article. It is not found necessarily in pure tin any more than it is necessarily found in gold foil; for it is well known that gold foil may be pure and yet not cohesive; and, if the property of cohesiveness does not depend upon the purity entirely, of the material, it must depend, more or less, upon the treatment to which the material is subjected during its manufacture.

Heretofore gold foil has absorbed almost the entire attention of foil manufacturers, while tin foil has been made and thrown into the market merely, it would seem, to make truthful the statement common to all circulars, that "So and So manufactures dentists' foil of all kinds." In manufacturing a superior article of tin foil, manufacturers would be advancing greatly their own interests, and also the interests ef their patrons, for the latter would willingly give much more for a good article than they are now paying for an impure, improperly prepared and almost worthless stuff, commonly sold at the dental depots. But, to return: a good article at hand, it should not be handled more than is absolutely needful, in fact, should be treated as carefully as gold foil. The common method of rolling into a rope and cutting off in short pieces, is convenient, but has a tendency to destroy the integrity of the foil and render it less cohesive. I prefer to fold the coil carefully in strips of from two to three or more layers in thickness, and pack as with gold foil, folded in the same manner. It is often convenient to cut these strips into little blocks. An annealing pan, kept at a comparatively low temperature, is a valuable accessory while packing the tin. A water bath (the temperature of which cannot possibly exceed 212° F.) is, perhaps, preferable to the pan. The most desirable instruments are those having broad working ends, with well defined and sharp serrations—Butler's condensers, for example.—*Canada Journal of Dental Science.*

